# The Value of IgM Memory B-Cells in the Assessment of Splenic Function in Childhood Cancer Survivors at Risk for Splenic Dysfunction: A DCCSS-LATER Study

**DOI:** 10.1155/2023/5863995

**Published:** 2023-10-20

**Authors:** Bente M. Houtman, Iris Walraven, Elke de Grouw, Richard W. M. van der Maazen, Leontien C. M. Kremer, Eline van Dulmen-den Broeder, Marry M. van den Heuvel-Eibrink, Wim J. E. Tissing, Dorine Bresters, Helena J. H. van der Pal, Andrica C. H. de Vries, Marloes Louwerens, Margriet van der Heiden-van der Loo, Sebastian J. C. Neggers, Geert O. Janssens, Nicole M. A. Blijlevens, Annechien J. A. Lambeck, Frank Preijers, Jacqueline J. Loonen

**Affiliations:** ^1^Department of Hematology, Center of Expertise for Cancer Survivorship, Radboud University Medical Center, Nijmegen, Netherlands; ^2^Department for Health Evidence, Radboud University Medical Center, Nijmegen, Netherlands; ^3^Department of Laboratory Medicine—Radboudumc Laboratory of Diagnostics, Radboud University Medical Center, Nijmegen, Netherlands; ^4^Department of Radiotherapy, Radboud University Medical Center, Nijmegen, Netherlands; ^5^Princess Máxima Center for Pediatric Oncology, Utrecht, Netherlands; ^6^Wilhelmina Children's Hospital, University Medical Center, Utrecht, Netherlands; ^7^Department of Pediatric Oncology, Emma Children's Hospital, University of Amsterdam, Amsterdam, Netherlands; ^8^Department of Pediatric Oncology/Hematology, Amsterdam University Medical Center, Amsterdam, Netherlands; ^9^Department of Pediatric Oncology, Sophia Children's Hospital, Erasmus Medical Center, Rotterdam, Netherlands; ^10^Department of Pediatric Oncology/Hematology, Beatrix Children's Hospital/University of Groningen/University Medical Center Groningen, Groningen, Netherlands; ^11^Department of Internal Medicine, Leiden University Medical Center, Leiden, Netherlands; ^12^Department of Medicine, Section Endocrinology, Erasmus Medical Center, Rotterdam, Netherlands; ^13^Department of Radiation Oncology, University Medical Center Utrecht, Utrecht, Netherlands; ^14^Department of Hematology, Radboud University Medical Center, Nijmegen, Netherlands; ^15^Department of Laboratory Medicine, University Medical Center Groningen, Groningen, Netherlands; ^16^Department of Laboratory Medicine—Laboratory for Hematology, Radboud University Medical Center, Nijmegen, Netherlands

## Abstract

**Background:**

Childhood cancer survivors (CCS) who received radiotherapy involving the spleen or total body irradiation (TBI) might be at risk for splenic dysfunction. A comprehensive screening test for examining splenic dysfunction is lacking.

**Objective:**

We investigated whether IgM memory B-cells could be used to assess splenic dysfunction in CCS who received a splenectomy, radiotherapy involving the spleen, or TBI.

**Methods:**

All CCS were enrolled from the DCCSS-LATER cohort. We analyzed differences in IgM memory B-cells and Howell–Jolly bodies (HJB) in CCS who had a splenectomy (*n* = 9), received radiotherapy involving the spleen (*n* = 36), or TBI (*n* = 15). IgM memory B-cells < 9 cells/*µ*L was defined as abnormal.

**Results:**

We observed a higher median number of IgM memory B-cells in CCS who received radiotherapy involving the spleen (31 cells/*µ*L, *p*=0.06) or TBI (55 cells/*µ*L, *p* = 0.03) compared to CCS who received splenectomy (20 cells/*µ*L). However, only two CCS had IgM memory B-cells below the lower limit of normal. No difference in IgM memory B-cells was observed between CCS with HJB present and absent (35 cells/*µ*L vs. 44 cells/*µ*L).

**Conclusion:**

Although the number of IgM memory B-cells differed between splenectomized CCS and CCS who received radiotherapy involving the spleen or TBI, only two CCS showed abnormal values. Therefore, this assessment cannot be used to screen for splenic dysfunction.

## 1. Introduction

An increasing population of childhood cancer survivors (CCS) is affected by late-effects due to cancer treatment [1–3]. One of these late effects is splenic dysfunction after splenectomy, radiotherapy involving the spleen, or total body irradiation (TBI) [4]. Splenic dysfunction is associated with an increased risk for severe infections [5, 6]. Most severe infections occur within the first two years after splenectomy, with a prevalence of 2%–8% [6, 7]. However, the elevated risk remains lifelong and is associated with a high mortality rate of 50%–70% [4–6, 8].

To prevent severe infections in patients with splenic dysfunction, several guidelines have been developed. However, these guidelines are not evidence based. The guidelines advise preventive measures, such as vaccinations, use of antibiotics, and caution in situations with a high-infection risk for CCS who had a splenectomy and CCS who had radiotherapy involving the spleen or TBI as part of their cancer treatment [9, 10]. Although a radiotherapy mean dose constraint of 10 gray to the spleen has been suggested [11], a clear cutoff for radiation dose and exposed splenic volume is lacking for radiotherapy involving the spleen, leading to discordance between guidelines, and possibly to under- or overprotection of these CCS [4, 9, 10, 12]. A screening test for splenic dysfunction would provide more clarity for this risk. However, a reliable and easily applicable test is not widely available for surveillance on splenic dysfunction.

The currently available tests to assess splenic function focus on the filtering function of the spleen, and thus provide no information about the immunological function [13]. Furthermore, most of the available tests are expensive, laborious, user-dependent, and not widely available [13–15]. Counting of Howell–Jolly bodies (HJB) on peripheral blood smear is one of the most commonly used methods to assess splenic function. Even though the presence of HJB is very suggestive for splenic dysfunction, testing for HJB is not very sensitive for predicting splenic function [16]. Therefore, a more readily applicable, sensitive and specific test to determine the immunological function of the spleen would be highly appropriate for this population.

IgM memory B-cells (CD27^+^IgD^+^IgM^+^) might give an indication of the immunological function of the spleen [16]. This cell group is mainly located in the marginal zone of the spleen, where these cells function as a first line of defense, especially against encapsulated bacteria [17–20]. The level of IgM memory B-cells is reduced in splenectomized patients, and this reduction is associated with an increased risk for pneumococcal infections [21–24]. The effect of radiotherapy involving the spleen on the level of IgM memory B-cells in CCS has not been studied before.

The aim of the current study is to compare the level of IgM memory B-cells in CCS after splenectomy, radiotherapy involving the spleen, and TBI, and thereby investigate whether IgM memory B-cell counts can be used to inform about splenic dysfunction by using observational data from a nationwide Dutch cohort. We also investigated the relation between the presence of HJB and the number of IgM memory B-cells.

## 2. Materials and Methods

### 2.1. Study Population

In this cross-sectional study, patients were included from the nationwide DCCSS-LATER cohort. This cohort consists of 5-year CCS, who were diagnosed with a malignancy according to the third edition of the International Classification of Childhood Cancer (ICCC3) [25] before the age of 18 years, between January 1^st^, 1963 and December 31^st^, 2001. All CCS were living in the Netherlands at time of diagnosis and were treated in one of seven Dutch pediatric oncology/hematology centers. For all participants, data were collected from patient files, questionnaires, and during a visit at the centers of expertise for late effects of childhood cancer (LATER outpatient clinics).

The current study included all CCS ≥ 18 years old at the time of inclusion who participated in the LATER 2 study as described by Feijen et al. [26], who received treatment affecting splenic function, and who were treated at the Radboud University Medical Center Nijmegen (Radboudumc) or at the University Medical Center Groningen (UMCG).

### 2.2. Treatment Groups

For this study, treatments affecting splenic function were categorized into three groups: (1) splenectomy, (2) radiotherapy involving the spleen, and (3) TBI as part of cancer treatment. Group 2 includes all CCS who received radiotherapy involving the spleen, including radiotherapy over the left hemiabdomen, left kidney, inverted-Y, para-aortic, total node, and (total) abdominal irradiation.

### 2.3. Flow Cytometry

Eight-color flow cytometric analysis was performed to detect total B-cells and B-cell subsets, including IgM memory B-cells (CD27^+^IgD^+^IgM^+^). Whole blood was drawn into standard ethylenediaminetetraacetic acid containing collection tubes. Erythrocytes were lysed before staining the peripheral blood cells for 15 min at 4°C with a panel of monoclonal antibodies (mAbs) at optimal concentrations. Total B-cells and B-cell subsets were calculated as absolute number of cells per *µ*L. Gating strategies for the detection of IgM memory B-cells are depicted in Figure S1.

At the Radboudumc the following mAbs were used: CD19-ECD (J3.119), CD20-PacBlue (B9E9), CD21-PE (B-Ly4), CD27-PE-Cy7 (1A4CD27), CD27-PE-Cy5.5 (1A4CD27), CD38-PE-Cy7 (LS198-4-3), CD45-BV510 (HI30), polyclonal IgM-FITC, polyclonal IgD-FITC, and IgM-PE (SA-DA4). Assays were performed on Navios Flow Cytometer (Beckman Coulter) and analyzed with Kaluza analysis software (version 2.1.3; Beckman Coulter).

At the UMCG the following mAbs were used: CD19-PerCP-Cy5.5 (SJ25C1), CD27-APC (L128), CD38-APC-H7 (HB7), antihuman IgD-PE (IA6-2), polyclonal antihuman IgM-FITC, antihuman IgG-BV510 (G18-145), CD21-Horizon V450 (B-ly4), and CD24-PeCy7 (ML5). Analyses were performed on FACSCanto II Flow Cytometer (BD Biosciences) and analyzed with FACSDiva software (version 7.0; BD Biosciences).

Flowcytometric analyses of both centers were compared, and similar results were found. Absolute number of total B-cells and B-cell subsets were compared to age-matched reference values [27]. The 5^th^ percentile of IgM memory B-cells in individuals >16 years old of 9 cells/*µ*L was used as the lower limit of normal.

### 2.4. Immunoglobulins

Total IgG, IgA, and IgM were determined on a Cobas c501 analyzer (Roche Diagnostics, Mannheim, Germany) with an immunoturbidimetric assay at the Radboudumc in Nijmegen (Tina-quant IgA Gen.2, Tina-quant IgG Gen.2, Tina-quant IgM Gen.2; Roche Diagnostics, Mannheim, Germany). Normal values as defined by the manufacturer were used to determine if results were within the normal range.

### 2.5. Howell–Jolly Bodies

Blood was analyzed for the presence of HJB. Blood films were stained with May–Grünwald Giemsa staining. Presence of HJB was assessed by an independent technician at the Radboudumc.

### 2.6. Statistical Analysis

Descriptive statistics were used to investigate the normality of the distributions. For the analysis, CCS were divided according to the treatment group. A Mann–Whitney *U* test was performed to evaluate differences in the level of IgM memory B-cells, as well as differences in the total B-cells and other B-cell subsets and immunoglobulins. A Mann–Whitney *U* test was performed to analyze the difference in IgM memory B-cells between CCS with and without detectable HJB in the blood film. A Pearson correlation coefficient was performed to evaluate correlations between IgM memory B-cells, age at treatment, age at study enrollment, and IgM. Data were analyzed using SPSS (version 25; IBM SPSS Statistics, Armonk, NY) and R (version 3.5.3; R foundation, Vienna, Austria). Significance was considered with a probability of <0.05.

## 3. Results

### 3.1. Patients

**Figure 1:**
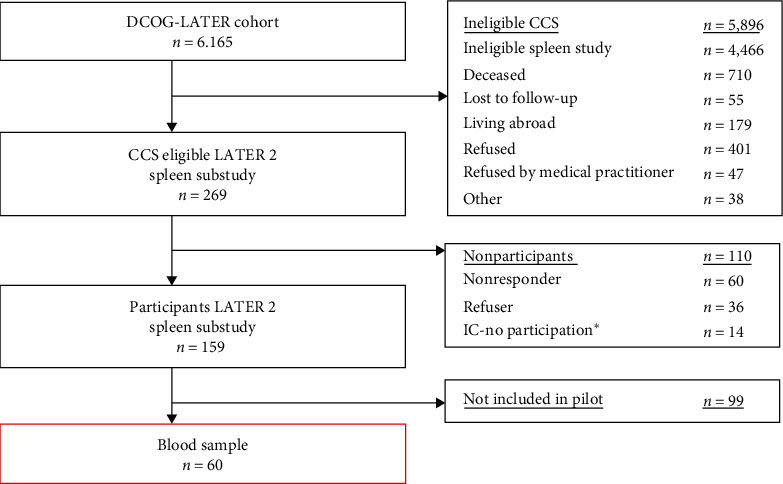
Flowchart. ^*∗*^IC – no participation: gave consent, however did not participate.

The DCCSS-LATER cohort consists of 6,165 CCS. From the 269 CCS who were eligible for the SPLEEN substudy, 159 (59%) CCS were included. Blood samples for flowcytometric analysis were taken in a subgroup of 60 CCS (38%) who were included for the DCCSS-LATER study at the UMCG and Radboudumc (Figure 1).

Of the included CCS, 9 (15%) had a splenectomy, whereas 36 (60%) were treated with radiotherapy involving the spleen, and 15 (25%) with TBI.  Table 1 shows the patient characteristics. All splenectomized CCS were diagnosed with Hodgkin Lymphoma. Compared to the other groups, more splenectomized CCS were treated before 1990, time to study enrollment was longer, and age at follow-up was higher. In the radiotherapy involving the spleen group most CCS were diagnosed with nephroblastoma (*n* = 20, 56%). Age at diagnosis was younger compared to the other treatment groups. In the TBI group most CCS were diagnosed with leukemias (*n* = 14, 93%). All patients in this group received a hematopoietic stem cell transplantation.

### 3.2. IgM Memory B-Cells

**Figure 2:**
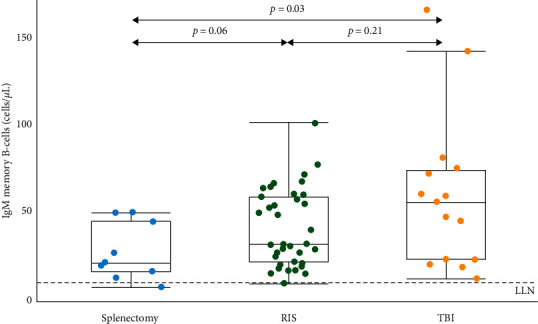
Distribution of IgM memory B-cells in cells/*µ*L by treatment affecting splenic function. Boxplots show the median as the horizontal line in the middle of the box, the upper and lower end of the box represent the 25^th^ and 75^th^ percentile, and the whiskers represent the smallest and largest values that are not outliers. LLN: lower limit of normal (9 cells/*µ*L).

Absolute numbers of IgM memory B-cells (CD27^+^IgD^+^IgM^+^) were determined in peripheral blood of CCS who received splenectomy, radiotherapy involving the spleen, or TBI.  Figure 2 shows the median number of IgM memory B-cells stratified per treatment group. CCS with splenectomy had a significantly lower number of IgM memory B-cells compared to CCS who had TBI as part of treatment (median 20 cells/*µ*L, IQR: 14–47 cells/*µ*L vs. 55 cells/*µ*L, IQR: 22–75 cells/*u*L, *p*=0.03). IgM memory B-cells of CCS with splenectomy were not significantly different from CCS treated with radiotherapy involving the spleen (median 20 cells/*µ*L, IQR: 14–47 cells/*µ*L vs. 31 cells/*µ*L, IQR: 20–59 cells/*µ*L, *p*=0.06). IgM memory B-cell values were within the range of all splenectomized CCS in 22 (61.1%) CCS with radiotherapy involving the spleen as part of treatment, and 7 (46.7%) CCS with TBI. Two CCS had IgM memory B-cells below the lower limit of normal. One had a splenectomy, and one had radiotherapy involving the spleen as part of cancer treatment.

To put the results of the IgM memory B-cells in context and to examine if the CCS had potential immune deficiencies, total B-cells and other B-cell subsets, as well as immunoglobulins were analyzed. These results are shown in Table S1. Though some differences between groups were found, most CCS had values of B-cells and immunoglobulins within the normal range with some values just below the lower limit of normal, which had no clinical relevance. IgM was on the lower side of normal in all treatment groups and was lower in CCS with splenectomy compared to the other treatment groups. However, no correlation with IgM memory B-cells was found. A significant positive correlation was found between IgM memory B-cells and age at enrollment (*r* = −0.30, *p* = 0.02). Age at treatment was not correlated to the number of IgM memory B-cells.

The CCS who had chronic graft versus host disease (cGvHD) had no deficiencies in B-cells or immunoglobulins. No between group differences were found for CCS with an allogeneic hematopoietic stem cell transplantation who had cGvHD compared to no cGvHD.

### 3.3. Howell–Jolly Bodies

The presence of HJB in peripheral blood was assessed in 55 (92%) CCS. HJB were detected in 3 out of 7 (43%) CCS who had a splenectomy and 2 out of 12 (17%) CCS with TBI as part of treatment. No HJB were detected in the CCS treated with radiotherapy involving the spleen. There was no significant difference in the level of IgM memory B-cells between CCS in whom HJB were detected compared to the CCS in whom HJB were not detected (median 35 cells/*µ*L, IQR: 20–58 cells/*µ*L vs. median 44 cells/*µ*L, IQR: 20–77 cells/*µ*L).

## 4. Discussion

This is the first study to investigate the absolute number of IgM memory B-cells, a potential immunological marker for splenic function, across CCS who received different treatments potentially affecting splenic function. We found a tendency towards a higher number of IgM memory B-cells in CCS treated with radiotherapy involving the spleen and TBI compared to the splenectomized CCS, with the highest median number in the TBI group. This supports our hypothesis that a lower level of IgM memory B-cells could be an indicator for reduced splenic function, since TBI doses are generally low in children (<12 gray). Doses used for abdominal irradiation are generally higher, which would likely have more impact on the splenic function, and thus result in a lower amount of IgM memory B-cells. Splenectomy would logically correspond with the lowest amount of IgM memory B-cells. Most CCS treated with radiotherapy involving the spleen and TBI had a number of IgM memory B-cells that fell within the range found in splenectomized CCS. This could indicate that even though the overall number of IgM memory B-cells is higher in the radiotherapy involving the spleen and TBI group, a substantial part of these CCS have a splenic function comparable to splenectomized CCS. However, when comparing the results to the reference values used by the participating centers, only two CCS in the study had abnormally low IgM memory B-cells. Thus, it seems that even though IgM memory B-cells differentiate somewhat between splenectomized and irradiated CCS, they cannot be used to differentiate these CCS from the healthy population.

Though some differences between treatment groups were found for total B-cells, B-cell subsets and immunoglobulins, these differences are not clinically relevant, since the values found were within the limits of normal. CCS who had a splenectomy had significantly lower IgM compared to the CCS who received radiotherapy involving the spleen, but not compared to CCS who received TBI. This is most likely due to a lack of power. Since IgM memory B-cells produce IgM, this might explain the lower value of IgM in the splenectomized CCS [17, 19]. However, no significant correlation was found between IgM memory B-cells and IgM. In general, groups were too small to draw conclusions regarding the immunoglobulins. Other factors, such as age at treatment and cGvHD, were not associated with the number of IgM memory B-cells. Though a significant correlation between IgM memory B-cells and age at assessment was present, this might be due to differences within the study cohort. All CCS with splenectomy were diagnosed with Hodgkin Lymphoma and were generally older than the CCS in the other treatment groups. Thus it seems that this association is more likely based on the diagnosis and treatment than on age. Previous research did not find a decline in IgM memory B-cells with age [28, 29].

In our study HJB were detected in only half of the splenectomized patients, indicating that the absence of HJB does not give a reliable indication of splenic function. This finding is consistent with other studies [6, 13, 14]. Also, the presence of HJB did not correspond with a lower level of IgM memory B-cells, though groups were small. Since it has been shown that this method is not sensitive, it is very questionable whether this should be used for the screening purposes.

Several studies investigated the level of IgM memory B-cells in splenectomized patients and showed a reduction compared to healthy individuals [21–24]. Only two studies reported absolute numbers of IgM memory B-cells in patients splenectomized because of traumatic rupture of the spleen [30, 31]. Their results were comparable with the results of splenectomized CCS in our study. Both studies demonstrated a significant difference in the median and mean values compared to the healthy individuals. However, these studies did not report on overlap of IgM memory B-cells between healthy and splenectomized individuals, thus this does not indicate that IgM memory B-cells can accurately differentiate between these groups.

From these previous studies and our results, it seems that the number of IgM memory B-cells is affected by the splenectomy, and in a lesser extent by radiotherapy involving the spleen. However, from our study it can be concluded that the number of IgM memory B-cells cannot be applied in the clinic to evaluate splenic function at the individual level, since it poorly differentiates individuals with splenic dysfunction from healthy individuals. Also, there is no widely used reference value for IgM memory B-cells available. Several studies have published outcomes for IgM memory B-cells in the healthy individuals [27, 29–33]. However, due to the small sample sizes, high variability of IgM memory B-cells in the healthy population, and differences in the flow-cytometric analyses, these results cannot be compiled into a reference value for IgM memory B-cells.

For the indication of potential splenic dysfunction in CCS it might be better to define a radiation dose cutoff as long as no reliable diagnostic test is available. However, the dose–response relationship between radiation dose and the degree of splenic dysfunction is still unclear. Though most guidelines recommend preventive measures for CCS who received ≥40 gray to the spleen, the risk might be increased at lower radiation doses as well [4, 11].

Strengths of this study are the completeness of data in this cohort regarding cancer and treatment related information. The follow-up time of the cohort was long, which made it possible to analyze the long-term consequences of splenectomy and irradiation involving the spleen on IgM memory B-cells. Another strength is the use of the absolute number of IgM memory B-cells, since this is a more robust measure that is not influenced by the fluctuations in other cell groups.

Limitations of this study are the limited number of included CCS, lack of healthy controls, and the lack of data regarding the radiotherapy dose and field exposing the spleen. To establish a good reference sample, a very large sample of healthy controls is necessary due to the heterogeneity of IgM memory B-cells in the general population. We did not have the means to include such a large group of healthy controls. We also did not have data regarding severe infections in these CCS, which did not allow to correlate the findings regarding the number of IgM memory B-cells to the clinic.

## 5. Conclusion

In conclusion, we found that CCS who had a splenectomy had the lowest absolute number of IgM memory B-cells, followed by radiotherapy involving the spleen and finally TBI. However, it seems that IgM memory B-cells cannot differentiate asplenic or potentially hyposplenic CCS from the healthy population, and therefore IgM memory B-cells cannot be used to screen for splenic dysfunction at an individual level in clinical practice. Future research should focus on the radiation dose that is associated with splenic dysfunction to better identify CCS at risk.

## Figures and Tables

**Table 1:** Patient characteristics.

Characteristics	Study cohort *n* = 60	Splenectomy *n* = 9	Radiotherapy involving spleen *n* = 36	TBI *n* = 15
	*n* (%)	*n* (%)	*n* (%)	*n* (%)
Sex				
Male	36 (60.0)	7 (77.8)	18 (50.0)	11 (73.3)
Female	24 (40.0)	2 (22.2)	18 (50.0)	4 (26.7)
Primary cancer diagnosis				
Leukemias, myeloproliferative diseases, and myelodysplastic diseases	14 (23.3)	0 (0)	0 (0)	14 (93.3)
Hodgkin lymphoma	16 (26.7)	9 (100)	7 (19.4)	0 (0)
Nonhodgkin lymphoma	1 (1.7)	0 (0)	0 (0)	1 (6.7)
Neuroblastoma and other peripheral nervous cell tumors	6 (10.0)	0 (0)	6 (16.7)	0 (0)
Nephroblastoma	20 (33.3)	0 (0)	20 (55.6)	0 (0)
Rhabdomyosarcoma	2 (3.3)	0 (0)	2 (5.6)	0 (0)
Germ cell tumors, trophoblastic tumors, neoplasms of gonads	1 (1.7)	0 (0)	1 (2.8)	0 (0)
Age at diagnosis (year), median (IQR)	6.3 (3.3–10.3)	12.7 (8.8–13.2)	4.8 (2.7–8.2)	6.5 (3.6–12.0)
0–4	23 (38.3)	0 (0)	18 (50.0)	5 (33.3)
5–9	21 (35.0)	3 (33.3)	12 (33.3)	6 (40.0)
10–17	16 (26.7)	6 (66.7)	6 (16.7)	4 (26.7)
Treatment period				
1970–1979	8 (13.3)	1 (11.1)	7 (19.4)	0 (0)
1980–1989	22 (36.7)	6 (66.7)	10 (27.8)	6 (40.0)
1990–1999	25 (41.7)	2 (22.2)	17 (47.2)	6 (40.0)
2000–2009	5 (8.3)	0 (0)	2 (5.6)	3 (20.0)
Overall treatment modality				
Radiotherapy and surgery	3 (5.0)	2 (22.2)	1 (2.8)	0 (0)
Radiotherapy and chemotherapy	19 (31.7)	0 (0)	5 (13.9)	14 (93.3)
Radiotherapy and chemotherapy and surgery	38 (63.3)	7 (77.8)	30 (83.3)	1 (6.7)
HSCT				
Autologous	4 (6.7)	0 (0)	2 (5.6)	2 (13.3)
Allogeneic	13 (21.7)	0 (0)	0 (0)	13 (86.7)
No HSCT	42 (70.0)	9 (100.0)	33 (91.7)	0 (0)
Unknown	1 (1.7)	0 (0)	1 (2.8)	0 (0)
Chronic graft versus host disease				
Yes	5 (8.3)	0 (0)	0 (0)	5 (33.3)
No	55 (91.7)	9 (100)	36 (100)	10 (66.7)
Time from primary cancer diagnosis to study enrollment (year), median (min–max)	28.2 (17.1–45.8)	31.6 (25.3–40.2)	25.8 (17.6–45.8)	27.0 (17.1–37.7)
17–29	32 (53.3)	2 (22.2)	20 (55.6)	10 (66.7)
≥30	28 (46.6)	7 (77.8)	16 (44.4)	5 (33.3)
Age at study enrollment (year), median (min–max)	36.4 (21.7–51.5)	44.3 (39.7–49.2)	34.5 (21.7–48.3)	33.1 (26.2–45.9)
18–29	14 (23.3)	0 (0)	11 (30.6)	3 (20.0)
30–39	24 (40.0)	2 (22.2)	14 (38.9)	8 (53.3)
≥40	22 (36.7)	7 (77.8)	11 (30.6)	4 (26.7)

TBI, total body irradiation; HSCT, hematopoietic stem cell transplantation; IQR, interquartile range.

## Data Availability

The data underlying this article were provided by the DCCSS-LATER Consortium under license. Data will be shared on request to the corresponding author with permission of the DCCSS-LATER Consortium.

## Abbreviations

CCS: Childhood cancer survivors
TBI: Total body irradiation
DCCSS-LATER: Dutch childhood cancer survivor study long-term effects after childhood cancer
HJB: Howell–Jolly bodies
mAbs: Monoclonal antibodies
cGvHD: chronic graft versus host disease.

## Supplementary Materials

Supplementary Materials Figure S1: flowcytometry results with exemplary gating strategy to identify the IgM memory B-cells. (A) Gating strategy as performed at UMCG. (B) Gating strategy as performed at Radboudumc. Table S1: absolute numbers of B-cells and immunoglobulins in the peripheral blood, median (range).
