# Supplementary material for: The Value of IgM Memory B-Cells in the Assessment of Splenic Function in Childhood Cancer Survivors at Risk for Splenic Dysfunction: A DCCSS-LATER Study
Source: J Immunol Res. 2023 Oct 20;2023:5863995. doi: 10.1155/2023/5863995 (PMC10611543; doi:10.1155/2023/5863995)
Supplement: Supplementary Materials — Figure S1: flowcytometry results with exemplary gating strategy to identify the IgM memory B-cells. (A) Gating strategy as performed at UMCG. (B) Gating strategy as performed at Radboudumc. Table S1: absolute numbers of B-cells and immunoglobulins in the peripheral blood, median (range). [file 5863995.f1.docx]

**Supplementary**

|  | **Splenectomy (n=9)** | | **Radiotherapy involving the spleen (n=36)** | | **Total body irradiation (n=15)** | |
| --- | --- | --- | --- | --- | --- | --- |
| **Total B-cells (CD45^+^ CD19^+^)** | 388 | (89-857) | 239 | (65-941)^b^ | 342 | (55-860) |
| **Transitional B-cells (CD27^-^ CD21^+^ CD38^++^ IgM^++^)** | 5 | (1-39) | 5 | (0-21) | 4 | (0-22) |
| **Naive B-cells (CD27^-^ IgM^+^ IgD^+^ CD38^+^)** | 298 | (62-741) | 146 | (31-763) | 218 | (35-682) |
| **Memory B-cells** | 54 | (15-99) | 66 | (22-180) | 88 | (22-180) |
| **IgM memory B-cells (CD27^+^ IgM^++^ IgD^+^)** | 20 | (6-49)^b^ | 31 | (8-101) | 55 | (11-166) |
| **IgM only B-cells (CD27^+^ IgM^+^ IgD^-^)** | 1 | (0-4)^b^ | 2 | (0-10)^b^ | 4 | (0-71) |
| **Class-switched memory B-cells (CD27^+^ IgM^-^ IgD^-^)** | 22 | (8-51) | 24 | (5-120) | 29 | (6-103) |
| **IgG**^1^ | 10.8 | (8.7-12.3)^b^ | 9.9 | (5.7-15.4)^b^ | 9.2 | (7.0-13.7) |
| **IgM**^1^ | 0.4 | (0.2-1.2)^a^ | 0.9 | (0.3-2.1) | 0.8 | (0.2-2.0) |
| **IgA**^1^ | 2.3 | (1.5-2.7) | 2.0 | (0.7-4.8) | 2.3 | (0.7-4.9) |

**Table S1.** Absolute numbers of B-cells and immunoglobulins in peripheral blood, median (range).

^1^ % (range)

^a^ P ≤0.05 compared to radiotherapy involving the spleen;

^b^ P ≤0.05 compared to total body irradiation;

**(A)**


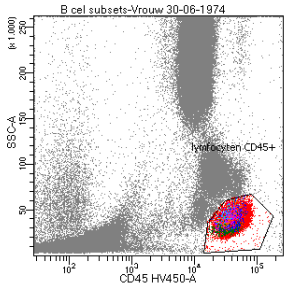

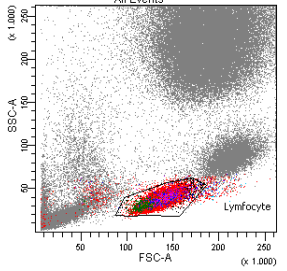

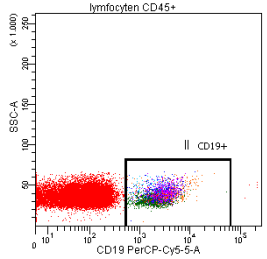

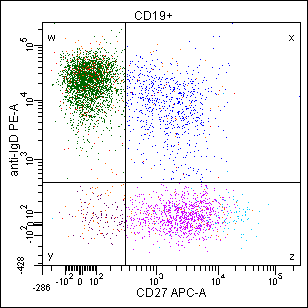

SS

SS

CD45

FS

CD19

SS

IgM

CD27

IgM memory B-cells

IgM memory B-cells

**(B)**

IgD

CD19

CD45

FS

SS

SS

SS

CD27

**Figure S1.** Flowcytometry results with exemplary gating strategy to identify IgM memory B-cells

(**A**) Gating strategy as performed at UMCG**.** (**B**) Gating strategy as performed at Radboudumc.
